# Microbial Profiles and Risk Factors of Preexisting Biliary Infection in Patients with Therapeutic Endoscopy

**DOI:** 10.1155/2019/1527328

**Published:** 2019-05-05

**Authors:** Hua-Qiang Ruan, Guo-Lin Liao, Peng Peng, Shi-Quan Liu, Chang-Liang Wu, Jian-Fu Qin, Zhi-Hai Liang, Guo-Du Tang, Meng-Bin Qin, Jie-An Huang

**Affiliations:** ^1^Department of Gastroenterology, The Second Affiliated Hospital of Guangxi Medical University, Nanning 530007, China; ^2^Department of Gastroenterology, The First Affiliated Hospital of Guangxi Medical University, Nanning 530021, China

## Abstract

**Background:**

The bile infection may already exist before the administration of an interventional procedure, despite no clinical manifestations of cholangitis detected. Blood cultures remained negative even in more than half of the febrile cases with cholangitis. Risk factors associated with bacterial growth in bile before the intervention are not well defined. To establish the bacterial profiles isolated from the bile samples and to identify risk factors for bacterial colonization in the bile system.

**Methods:**

Individuals who underwent endoscopic retrograde cholangiopancreatography (ERCP) interventions were enrolled. Bile samples were aspirated and were immediately transferred into a sterile tube for storage.

**Results:**

Positive bile cultures were detected in 363 (38.0%) of 956 patients, including 322 benign diseases and 41 malignances. Of 363 positive cases, 351 (96.7%) were monoinfection and 12 (3.3%) multi-infection. *Escherichia coli* were the most common Gram-negative bacteria (210, 56.0%), followed by *Klebsiella pneumoniae* (45, 12.0%). *Enterococcus faecalis* represented the most common Gram-positive microorganism (19, 5.07%), while *Candida albicans* (11, 2.93%) were the dominant fungi. *Klebsiella pneumoniae* were more frequently detected in malignant diseases (*P* = 0.046). Age, previous ERCP history or OLT history, and CBD diameter were independent risk factors for positive cultures (*P* < 0.05) while preoperative jaundice drug therapy was the protective factor for bile infection (*P* < 0.05).

**Conclusion:**

Monomicrobial infection was dominant among all infections, and *Klebsiella pneumoniae* strains were more frequently isolated from patients with malignant diseases. To effectively manage patients who are at a high risk for bile infection, a detailed diagnosis and treatment plan for each case should be prepared.

## 1. Introduction

The bile duct is typically maintained sterile by the continuous flushing action of bile and the bacteriostatic effects of bile salts [1]. Under the conditions of normal bile flow, positive bile culture is not expected. However, bacteria could remain, colonize, and replicate in a relatively stagnant bile environment if a biliary obstruction exists resulting in the increase of pressure. Eventually, the bacteria would spread into the blood and cause systemic infections posing grave consequences.

Endoscopic retrograde cholangiopancreatography (ERCP) has gradually become an indispensable procedure in the diagnosis and treatment of many pancreaticobiliary disorders since its first introduction in the 1970s. Despite a safe and effective record for ERCP, endoscopists must have a thorough understanding of potential adverse events associated with the procedure. Cholangitis is a common adverse event linked to the ERCP procedure [2]. About 0.5% to 3% of ERCP cases [3–8] or 0.35% to 2.4% of ERCP cases in China [9, 10] developed cholangitis after the procedure.

Previous studies suggested incomplete drainage in an obstructed biliary system resulting from choledocholithiasis and incomplete stone clearance were the main risk factors for post-ERCP cholangitis (PEC) [11], especially after the contrast injection. Biliary pathological changes are often secondary to a bacterial colonization postprocedure [12]. The infection of the biliary tract causing bacteremia may manifest as a compatible clinical syndrome, and a blood culture isolate profile may reflect the original infection of cholangitis. However, the infection potentially exists before the ERCP procedure despite lacking the typical manifestations of cholangitis (jaundice, fever, and right upper quadrant pain) [13, 14]. Blood cultures remained negative in more than half of the febrile cases with cholangitis [15]. Currently, the risk factors for bacterial growth in bile before the intervention have not been clearly defined [16, 17].

The aim of this study is to establish the bacterial profiles isolated from the bile samples and their contributions to the underlying diseases. Furthermore, we investigate the risk factors for microbiological colonization in patients with different biliary diseases.

## 2. Methods

### 2.1. Patients

Patients who had various biliary or pancreatic disorders and received ERCP procedures between January 2012 and May 2018 at the First and Second Affiliated Hospitals of Guangxi Medical University (Nanning, China) were selected for this study. Patients were excluded if bile aspirations failed or bile culture data did not exist, cholecystectomy, under 18 years of age or in the event of incomplete clinical data. The study was approved by the Institutional Review Boards of Hospitals. A written informed consent was obtained from all participants.

### 2.2. Procedures

All ERCP procedures were administered by well-trained and experienced endoscopists, who could perform the procedures that have the ERCP difficulty at Grade 3 per the ERCP core curriculum [18].

The ERCP interventions were conducted using a therapeutic duodenoscope (TJF-260V; Olympus Optical, Tokyo, Japan). The selective cannulation was performed via the common bile duct (CBD) by using a guidewire or a standard catheter for cases with a preexisting sphincterotomy. All duodenoscopes were disinfected according to the guidelines and decontaminated, assessed by regular smear tests. Once the duodenoscope entered successfully and guidewire cannulation was established, bile was aspirated by inserting a single use, 5F, standard sphincterotome catheter into the bile duct before the injection of a contrast agent for the ERCP procedure. Approximately 2 to 8 mL of bile (average 4 mL) was collected and immediately transferred into a sterile tube.

### 2.3. Observational Index

The demographics and clinical information before the cholangiopancreatography were collected including patients' gender and age, endoscopic diagnoses, previous ERCP history, preoperative jaundice drug therapy, Billroth II gastrectomy history, orthotopic liver transplantation (OLT) history, common bile duct diameter, and papilla types. Patterns of cannulation of univariate and multivariate analysis were performed to identify the independent risk factors of bacterial colonization in bile.

### 2.4. Statistical Analysis

Continuous variables were expressed as the mean and standard deviation (s.d.) or the median and IQR. Counts and percentages were determined if appropriate. The categorical variables were analyzed by Pearson's chi-square test or Fisher's exact test. In univariate analysis, the test level was unrestricted to 0.10 if the covariates exhibited a high level of significance. Multivariate regression analyses were used to identify various risk factors. Logistic regression models were employed to calculate odds ratios with 95% confidence intervals (CIs). A two-tailed *P* value < 0.05 was considered statistically significant (SPSS 22.0 for Windows, SPSS, Chicago, IL).

## 3. Results

### 3.1. General Characteristics

**Figure 1:**
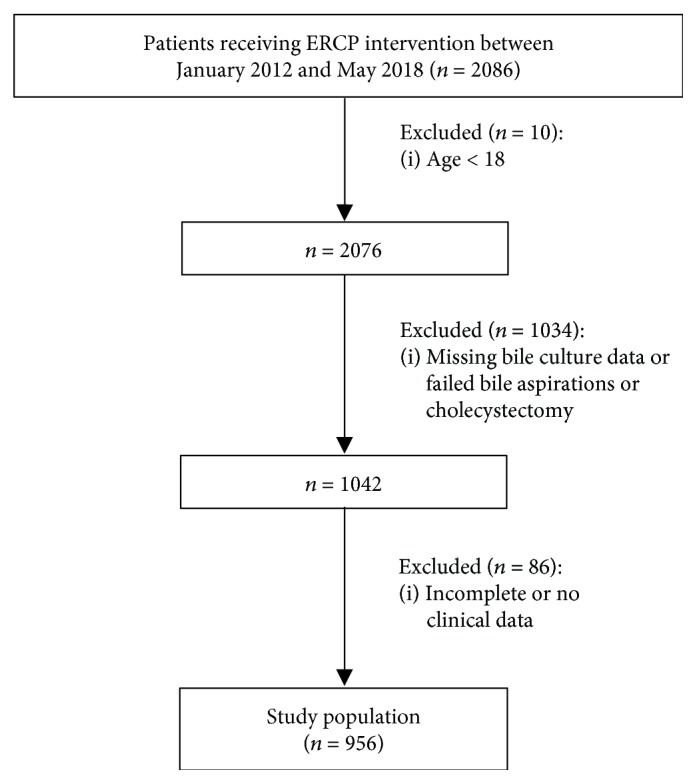
Patient selection flow chart.

This retrospective study initially screened 2086 consecutive patients who underwent ERCP in the two hospitals between January 2012 and May 2018. The information contained in medical charts, computerized records, and image studies was retrieved. Patients were excluded if under 18 years of age (*n* = 10), no bile culture data, failed bile aspirations or cholecystectomy (*n* = 1034), or incomplete clinical data (*n* = 86). Note that the bile collection is not generally recommended by gastroenterology societies due to being technically demanding, time consuming, or redundant in the presence of blood cultures. Thus, the bile collection is an option under the ERCP procedure and contingent upon various indications, resulting in no bile collections in a considerable number of patients with ERCP procedures [17]. In addition, patients were also excluded because of inadequate bile aspiration. A total of 956 patients were finally enrolled and analyzed (Figure 1). The mean age was 58.4 ± 15.0 years (range: 18-92 years), and 61.3% of the cohort were male. Among the 956 patients, 835 had benign diseases and 121 with malignances. All patients with acute cholangitis were treated with antibiotics prior to cholangiography.

The endoscopic diagnosis consisted of 694 cholelithiasis (72.6%), 121 malignant strictures (12.7%), 58 benign strictures (6.1%), 39 bile duct expansions for unknown reasons (4.1%), five pancreatic disorders (0.5%), 21 normal cholangiopancreatography (2.2%), nine clonorchiasis (0.9%), and nine other diseases (0.9%), which included six congenital choledochal cyst, two biliary fistulas after surgeries, and one sphincter of Oddi dysfunction (SOD) (Table 1).

### 3.2. Microbiological Characteristics in Bile Culture

Positive bile cultures were detected in 363 (38.0%) of 956 patients, including 322 with benign diseases and 41 with malignant diseases. Of 363 positive samples, 351 (96.7%) were single bacterial infection and 12 (3.3%) were mixed infection. Further, a total of 34 species and 375 strains of microorganisms were identified (Table 2). There were 298 strains of Gram-negative bacteria, 51 strains of Gram-positive bacteria, and 26 strains of fungi. Five strains of Multidrug-Resistant Organisms (MDRs) were identified including four *Acinetobacter baumannii* and one *Acinetobacter lwoffii*. Among the 298 Gram-negative strains, the most common ones were *Escherichia coli* (210, 56.0%), *Klebsiella pneumoniae* (45, 12.0%), *Pseudomonas aeruginosa* (8, 2.13%), and *Enterobacter cloacae* (8, 2.13%). The most common Gram-positive bacteria were *Enterococcus faecalis* (19, 5.07%) and *Enterococcus faecium* (14, 3.73%). The most common fungi were *Candida albicans* (11, 2.93%) and *Candida tropicalis* (7, 1.87%).

In addition, we investigated the four most prevalent microorganism distributions in benign and malignant diseases. A greater number of *Klebsiella pneumoniae* strains were detected in malignant diseases compared to those found in benign diseases (22.4% vs. 11.8%, *P* = 0.046), while there were no significant differences in the numbers of strains of *Escherichia coli* (58.7% vs. 51.2%, *P* = 0.456), *Enterococcus species* (10.9% vs. 19.5%, *P* = 0.175), and fungi (5.0% vs. 7.3%, *P* = 0.792) between benign and malignant disease groups (Table 3).

### 3.3. Risk Factors for Bacterial Colonization in Bile

Univariate analysis demonstrated that age, previous history of ERCP, previous orthotopic liver transplantation, preoperative jaundice drug therapy, various endoscopic diagnoses, common bile duct diameter, different papilla types, and patterns of cannulation were all risk factors for positive bile culture (*P* < 0.10) (Table 4).

The variables identified through the univariate analysis and additional factors selected based on clinical experience and the literature were included in a multivariate logistic regression analysis. Our multivariate analysis established age, previous history of ERCP, previous history of orthotopic liver transplantation, and common bile duct diameter as independent risk factors for positive bile culture (*P* < 0.05) (Table 5). Nevertheless, preoperative jaundice drug therapy is classified as a protective factor against positive bile culture (*P* < 0.05).

## 4. Discussion

A normal sterile and free flow biliary system is not a favorable environment for bacterial growth. However, bile secretion can be restricted or blocked within the bile duct due to biliary obstruction. The bacteria that are transferred into the bile duct through the duodenal papilla can reside in the bile duct under the restricted bile flow scenario, ultimately replicating, colonizing, and causing biliary infection with severe consequences [19].

In this study, we identified the microbial profiles in infected bile systems. The positive bile culture rate was 38.0% among the 956 patients analyzed. Gram-positive and Gram-negative bacteria accounted for 13.6% and 78.1%, respectively, with the remaining 7% fungi positive. *Escherichia coli* was the most common Gram-negative bacteria, followed by *Klebsiella pneumoniae*, *Pseudomonas aeruginosa*, and *Enterobacter cloacae*. *Enterococcus* species represented the most common Gram-positive bacteria. The composition of biliary pathogens was consistent with other studies, resembling the intestinal bacterial flora [14, 20]. In a large study cohort involving 509 consecutive individuals who underwent early laparoscopic cholecystectomy (within 72 hours) or percutaneous cholecystostomy, 171 (33.6%) of them tested bile culture positive. Gram-negative organisms accounted for 80.1% (137/171). Among them, *E. coli* was the most frequent isolate while *Enterococcus* was the most common Gram-positive sample [21]. Other independent studies reported 16% to 85% positive bile culture among different disease groups [14, 15, 17, 22–25].

The detected monomicrobial infection was more frequently (351/363, 96.7%) compared with multimicrobial infection (12/363, 3.3%). Kaya et al. [14] reported that a single bacterobilia accounted for 95% of infection. However, higher multi-infection rates compared to monoinfection rates were reported in other studies [17, 26]. Overprescription of antibiotics before hospitalization in China or suboptimal culture conditions may explain the differences. Monomicrobial infection, especially with Gram-negative bacterium, was frequently associated with patients requiring repeated ERCP interventions. This finding provides guidance to select an initial antibiotic regimen.

Published studies suggested a trend of higher infection rates of *Helicobacter spp*. in individuals with malignant bile duct diseases compared to normal controls or those with benign biliary diseases [27]. Thus, we analyzed the distribution of the identified organisms between our benign and malignant groups. In the benign pancreaticobiliary diseased group, *Escherichia coli* strains were dominant, followed by *Klebsiella pneumoniae* and *Enterococcus spp*. A similar trend was noted in the malignant group. These results may guide physicians to select more efficacious antimicrobials. However, the infection with *Klebsiella spp.* was significantly higher in malignant biliary diseases (*P* = 0.046, Table 3). Other studies [28, 29] also reported a higher culture rate of the *Klebsiella pneumoniae* strain among malignant diseases. *Klebsiella spp.* (mainly *Klebsiella pneumoniae*) is frequently found in human intestines and the upper respiratory tract causing opportunistic infections. The antibacterial effectiveness can be hampered by antibiotic resistance. It was noteworthy that malignant diseases appeared more frequently in individuals aged 60 years or older compared to those younger patients.

Unrestricted cancer growth deprives the body of proper nutrition, injures the mucosal barrier, compromises immunity function, and ultimately leads to infection. The microorganism profiles isolated from the bile samples of malignant diseases differed from those described in the Tokyo Guidelines and other studies of benign biliary disorders. This knowledge provides an alternative solution when selecting empirical antibiotics prior to the availability of a positive culture or after a negative culture.

The multivariate study identified old age as an independent risk factor for positive bile cultures. Mahafzah and Daradkeh reported that the percentage of positive cultures increased with age [30]. It is highly recommended that the elderly patients should be adequately evaluated for bile infection before the intervention.

This study found that previous ERCP history or OLT history was associated with positive cultures. A significantly higher bacteriobilia was observed in patients who required repeated interventions and orthotopic liver transplantation, as reported in a study cohort with 243 consecutive patients who underwent ERCP or percutaneous transhepatic cholangiography (PTC) [17]. Yun and Seo [31] suggested the bile of patients with laparoscopic cholecystectomy may contain microorganisms, particularly in those who received repeated ERCP. Interventions, like Oddi's sphincterotomy, breakdown the normal human defense mechanisms, decrease the pressure of the bile duct, and potentially cause a reverse fluid flow from the duodenum into the bile duct [9, 26]. The individuals who underwent these interventions might have acquired biliary tract motor dysfunction and are prone to biliary infection. Furthermore, the bile duct at anastomosis location after surgery like OLT could become narrowed, and the anatomical structures could be altered. With the reflux of bile increasing, biliary mucosa could experience inflammatory edema, ultimately generating a favorable environment for bacterial proliferation and colonization.

Common bile duct diameters at ≥12 mm were also determined as an independent risk factor, which has been suggested to be an important risk factor for post-ERCP cholangitis with biliary type sphincter of Oddi dysfunction [32], especially in type I. However, it remains unclear how CBD diameter triggers the positive bile culture before the ERCP interventions. Long-term disease courses, repeated inflammatory stimulations, large CBD stones and distal stricture caused by benign or malignant diseases may induce the bile duct obstruction or dilatation. Consequently, an increased biliary pressure traps and retains microorganisms within the stagnant bile.

We noted a higher tendency of positive bile culture in patients with papillary carcinoma in this analysis. Papillary carcinoma may cause malignant biliary obstruction as well as biliary infection, and a combination of both might have a profound effect on clinical outcomes, as well as the quality of life [33].

Interestingly, preoperative jaundice drug therapy was identified as an independent protective factor for the absence of infection. The alleviated jaundice therapy may improve biliary drainage and decrease the retention of organisms in the bile duct.

The use of prophylactic antibiotics before the ERCP procedure is not routinely required, according to the guidelines updated by the Digestive Endoscopy Branch of British Society of Digestion (BSG) in June 2009. A full antibiotic course is required when the treatment is aimed at achieving adequate biliary decompression by repeat interventions [34]. Currently, the third-generation cephalosporins or penicillin/*β*-lactamase inhibitors are recommended as the empirical treatment for biliary infections [35]. The bile culture is invaluable in the selection of efficacious and appropriate antibiotics for the treatment of biliary infections.

To avoid cross-transmission among different patients, the facilities and instruments were maintained at a sterilized status. We have followed stringent standards to conduct all ERCP procedures. We set the detection of microorganisms at a level of 10,000 per mL of bile as positive, and anything below this level was deemed negative [14, 17]. We are confident in the validity of the bile culture results. However, we could not completely exclude the possibility of bile sample contamination during which the duodenoscope was passed through the gastrointestinal tract.

Additional limitations include the following: an inherent selection bias associated with a retrospective study; the lack of confirmation of positive bile cultures by blood culture; and the need for multicenter different region-based studies.

In conclusion, our results show that 38% of patients with various biliary or pancreatic disorders had bacteriobilia. The most commonly isolated bacteria were Gram-negative bacteria including *Escherichia coli* and *Klebsiella pneumoniae.* Monomicrobial infection was more prevalent compared with multimicrobial infection. A higher percentage of *Klebsiella pneumoniae* strains was detected in the malignant diseases compared to the benign group. The identified risk factors associated with positive bile culture included old age, previous history of ERCP or OLT, and larger common bile duct diameter. A preoperative jaundice drug therapy was a factor associated with negative bile culture. An implication of our findings recommends that preoperative precaution should be adopted and a detailed management plan should be prepared in advance, considering about one-third of patients likely have had bile infection, previously. Bile samples should be collected for culture to confirm potential infection whenever possible.

## Figures and Tables

**Table 1:** Baseline demographics and endoscopic features of enrolled patients.

Study population	956
Mean age (years, s.d.)	58.4±15.0
Females (%)	38.70%
Benign diseases	835 (87.3%)
Malignant diseases	121 (12.7%)
*Endoscopic diagnoses*	
Normal cholangiopancreatography	21 (2.2%)
Cholelithiasis	694 (72.6%)
Malignant strictures	121 (12.7%)
Benign strictures	58 (6.1%)
Bile duct expansions for unknown reasons	39 (4.1%)
Pancreatic disorders	5 (0.5%)
Clonorchiasis	9 (0.9%)
Other diseases	9 (0.9%)

**Table 2:** Microbiological classification of positive bile culture.

Organisms	N	%
*Bacteria*		
Gram-positive	51	13.6
*Enterococcus spp.*	43	11.5
*Enterococcus faecalis*	19	5.1
*Enterococcus faecium*	14	3.7
*Enterococcus casselifavus*	7	1.9
Other *Enterococcus species*	3	0.8
*Streptococcus spp.*	6	1.6
*Streptococcus hemolyticus*	4	1.1
Other *Streptococcus species*	2	0.5
*Staphylococcus spp.*	2	0.5
Gram-negative	293	78.1
*Escherichia coli*	210	56.0
*Klebsiella spp.* (*3 K. oxytoca* included)	48	12.8
*Pseudomonas spp.*	9	2.4
*Enterobacter cloacae*	9	2.4
*Citrobacterium spp.*	4	1.1
*Serratia fonticola*	4	1.1
*Aeromonas hydrophila*	4	1.1
*Flavobacterium spp.*	2	0.5
*Proteobacteria spp.*	2	0.5
*Morganella Fulton*	1	0.3
*MDRs*		
*Acinetobacter baumannii*	4	1.1
*Acinetobacter lwoffii*	1	0.3
*Fungi*		
*Candida albicans*	11	2.9
*Candida tropicalis*	7	1.9
*Candida glabrata*	5	1.3
*Candida cornea*	3	0.8

**Table 3:** Bacterial composition between benign and malignant diseases.

Species	Benign (*n* = 322) (%)	Malignant (*n* = 41) (%)	*P*	Total (*n* = 363) (%)
*Escherichia coli*	189 (58.7)	21 (51.2)	0.456	210 (57.9)
*Klebsiella spp.*	38 (11.8)	10 (24.4)	**0.046**	48 (13.2)
*Enterococcus spp.*	35 (10.9)	8 (19.5)	0.175	43 (11.8)
*Fungi*	16 (5.0)	3 (7.3)	0.792	19 (5.2)

**Table 4:** Univariate analysis of risk factors for bile infection.

Factors	Positive culture (*n*)	Negative culture (*n*)	*χ* ^2^	*P* value
Gender				
Male	228	358	0.466	0.495
Female	135	235		
Age				
≥60 years	226	256	32.061	**<0.001**
<60 years	137	337		
Previous ERCP history				
Yes	145	129	35.558	**<0.001**
No	218	464		
Previous Billroth II history				
Yes	3	3	0.035	0.852
No	360	590		
Previous OLT				
Yes	13	5	7.716	**0.005**
No	359	588		
Preoperative jaundice drug therapy				
Yes	161	315	6.577	**0.010**
No	202	278		
Endoscopic diagnoses				
Normal cholangiopancreatography	1	20	17.719	**0.012**
Cholelithiasis	276	418		
Malignant strictures	41	80		
Benign strictures	27	31		
Bile duct expansions for UR	12	27		
Pancreatic disorders	1	4		
Other diseases	4	5		
Clonorchiasis	1	8		
Common bile duct diameters				
≥12mm	284	344	39.981	**<0.001**
<12mm	79	249		
Papilla types				
Normal	221	460	41.289	**<0.001**
Minor papilla	5	17		
Papillary diverticulum	104	77		
Papillary carcinoma	20	23		
Papillary fistula	13	16		
Patterns of cannulation				
Routine	353	557	5.412	**0.067**
Dual-guidewire	6	21		
Precut papillotomy	4	15		

OLT: orthotopic liver transplantation. Bile duct expansions for UR: unknown reasons. Papillary precut: a needle-like knife was used to cut layer by layer from 11 o'clock position of the papillary uplift highest point to papillary openings, or a needle-like knife was vertically used to pierce and fenestrate via the highest point of the papillary highest bump.

**Table 5:** Multivariate analysis of risk factors for positive bile culture.

Factors	Wald	OR	*P* value	95% CI
Preoperative jaundice drug therapy	5.267	0.704	**0.022**	0.521-0.950
Age	9.267	1.592	**0.002**	1.180-2.147
Common bile duct diameter	19.629	2.114	**<0.001**	1.518-2.944
Endoscopic diagnoses	5.819		0.561	
Cholelithiasis	0.246	0.475	0.620	0.025-8.957
Malignant strictures	0.654	2.414	0.419	0.285-20.450
Benign strictures	0.457	2.124	0.499	0.239-18.845
Bile duct expansions for unknown reasons	0.659	2.505	0.417	0.273-23.017
Pancreatic disorders	0.320	1.915	0.572	0.201-18.211
Other diseases	0.000	1.026	0.987	0.046-22.797
Clonorchiasis	1.905	5.889	0.168	0.475-73.035
Previous ERCP history	22.250	2.156	**<0.001**	1.567-2.967
Previous orthotopic liver transplantation	7.198	4.914	**0.007**	1.536-15.725
Papilla types	24.076		0.000	
Minor papilla	0.172	0.846	0.678	0.383-1.868
Papillary diverticulum	0.587	0.599	0.444	0.162-2.220
Tumor	2.960	2.082	**0.085**	0.903-4.799
Papillary fistula	0.171	1.254	0.679	0.429-3.662
Cannulation methods	3.486		0.175	
Dual-guidewire	1.685	2.167	0.194	0.674-6.962
Precut papillotomy	0.013	1.093	0.908	0.244-4.890

## Data Availability

The data used to support the findings of this study are available from the corresponding authors upon request.
